# Why Is ABI Effective in Detecting Vascular Stenosis? Investigation Based on Multibranch Hemodynamic Model

**DOI:** 10.1155/2013/185691

**Published:** 2013-09-05

**Authors:** Xiaoyun Li, Ling Wang, Chi Zhang, Shuyu Li, Fang Pu, Yubo Fan, Deyu Li

**Affiliations:** ^1^Key Laboratory for Biomechanics and Mechanobiology of Ministry of Education, School of Biological Science and Medical Engineering, Beihang University, Beijing 100191, China; ^2^State Key Laboratory of Virtual Reality Technology and Systems, Beihang University, Beijing 100191, China

## Abstract

The ankle-brachial index (ABI), defined as the ratio of systolic pressure in the ankle arteries and that in the brachial artery, was a useful noninvasive method to detect arterial stenoses. There had been a lot of researches about clinical regularities of ABI; however, mechanism studies were less addressed. For the purpose of a better understanding of the correlation between vascular stenoses and ABI, a computational model for simulating blood pressure and flow propagation in various arterial stenosis circumstances was developed with a detailed compartmental description of the heart and main arteries. Particular attention was paid to the analysis of effects of vascular stenoses in different large-sized arteries on ABI in theory. Moreover, the variation of ABI during the increase of the stenosis severity was also studied. Results showed that stenoses in lower limb arteries, as well as, brachial artery, caused different variations of blood pressure in ankle and brachial arteries, resulting in a significant change of ABI. Furthermore, the variation of ABI became faster when the severity of the stenosis increased, validating that ABI was more sensitive to severe stenoses than to mild/moderate ones. All these in findings revealed the reason why ABI was an effective index for detecting stenoses, especially in lower limb arteries.

## 1. Introduction

The partial occlusion of arteries due to stenotic obstruction is one of the most frequent cardiovascular diseases in human beings. The vascular stenosis frequently affects the blood pressure and flow of large and middle-sized arteries. Mathematical models based on one-dimensional flow equations are usually used to study the hemodynamic mechanism for the effects of vascular stenoses on cardiovascular system. Young et al. [1–3] developed finite element models including nonlinearities arising from geometry and material properties to analyze the characteristics of blood flow and pressure decrease caused by an arterial stenosis. Garcia et al. [4] and Singh and Shah [5] analyzed the decreases of instantaneous maximal transvalvular pressure in aortic stenosis using numerical models. Pralhad and Schultz [6] and Feng et al. [7] studied influences on blood cellular constituents and related blood diseases on molecular level. Models used in those studies were mostly applied to the analysis of hemodynamic effects of stenoses on blood pressure decreases or flow characteristics. However, few studies focused on the related detective indexes of the vascular stenosis, such as the ankle-brachial index (ABI).

**Figure 1:**
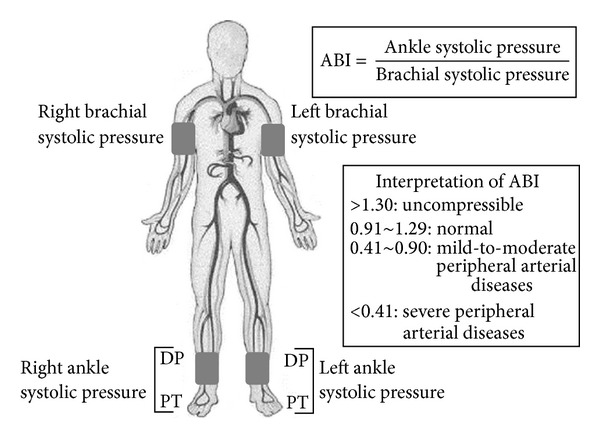
Measurement of the ankle-brachial index (ABI). DP indicates dorsalis pedis artery; PT, posterior tibial artery.

ABI, defined as the ratio of systolic pressure in the ankle arteries (the posterior tibial artery in this model) and systolic pressure in the brachial artery (Figure 1), was an important noninvasive measurement for the detection of arterial obstructive disease, especially for the lower extremity arterial stenosis [8–10]. The American College of Cardiology (ACC) and the American Heart Association (AHA) proposed to grade ABI into four levels [8], as shown in the diagram below. ACC/AHA had recommended the evaluation standards of normal ABI to be 0.91~1.30, and also a cutoff of 0.90 to define a low ABI value. The ABI value greater than 1.30 indicated that the vascular was uncompressible, suggesting that vascular calcification might have occurred. The ABI was generally unreliable for stenoses detection in such situations. The ABI value that ranged from 0.41 to 0.90 predicted the presence of mild-to-moderate stenoses. Severe stenoses were diagnosed with the ABI value less than 0.41 in clinical data. 

A lot of efforts have been made in the last decade to discuss the effectiveness and specificity of ABI in detecting arterial stenoses with clinical data. These researches have acquired a great number of insights into the sensitivity and specificity of ABI to diagnose peripheral arterial stenoses. Decrinis et al. [11] and Carter [12] found the sensitivity of ABI to be 94% and the specificity to be 100% by carrying out measurements in 146 limbs with angiographically documented arterial occlusive disease (AOD) and in 85 limbs without AOD. This strongly proved the validity of ABI in detecting arterial stenoses in lower extremity. Furthermore, many statistic studies were made to compare the sensitivity and the effectiveness of ABI in detecting arterial stenoses with different severities [13–15]. All experimental data agree to the point that the ABI measurement is a reliable noninvasive diagnostic method in assessing lower extremity arterial stenoses, which is alternative to conventional digital substraction angiography (DSA). The ABI value shows a decreasing tendency with increasing severity of the stenoses in patients with peripheral arterial stenotic diseases. However, these findings are just clinical regularities, lacking the mechanism study to interpret these situations.

The present study is thus performed to develop a computational multibranch model of the entire cardiovascular system including typical arterial units of lower/upper limb. The model is used to investigate the correlation between ABI and the stenosis in theory. The influences of stenoses located in different sites of the cardiovascular system on ABI are discussed in this paper, as well as the variation tendency of ABI value caused by the stenosis with the increasing severity. 

## 2. Methods

**Figure 2:**
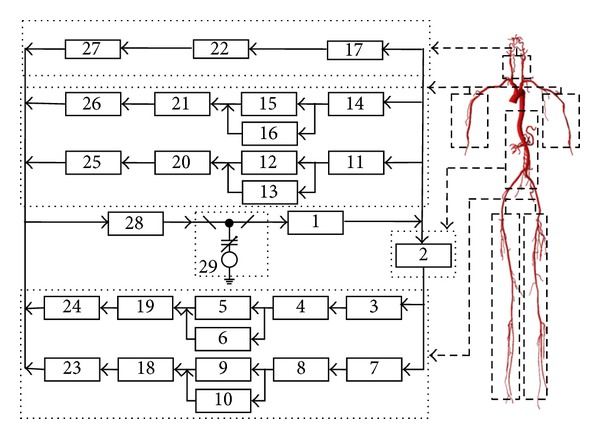
The electric analog circuit model of the entire cardiovascular system. Each component is comprised of a compliance variable *C*, a resistance *R*, and an inductance *L* (1: aorta (a); 2: thoracic and abdominal aortae (l); 3/7: left/right femoral artery (afl/afr); 4/8: left/right popliteal artery (apl/apr); 5/9: left/right posterior tibial artery (atl/atr); 6/10: other left/right lower limb arteries (all/alr); 11/14: left/right brachial artery (abl/abr); 12/15: left/right radial artery (arl/arr); 13/16: left/right ulnar arteries (aul/aur); 17: carotid artery (ac); 18/19: left/right lower limb capillaries (alpl/alpr); 20/21: left/right upper limb capillaries (aupl/aupr); 22: brain (acp); 23/24: left/right lower limb veins (vll/vlr); 25/26: left/right upper limb veins (vul/vur); 27: jugular veins (vc); 28: system vena (ve); 29: C_lv_ represents the compliance of the left ventricular). The effects of inertia for veins are not considered in this model.

A lumped parameter multibranch model with l7 arterial units was developed to simulate the pulse wave propagation of the cardiovascular system. Construction of the model was implemented based on a phenomenological characterization of hemodynamics using an electrical analog method. It was assumed that human body was completely symmetric and that the cardiovascular system could be represented by a lumped parameter model. Another assumption was that the blood was a Newtonian fluid and that the dispersed arterial networks could be modeled using linear circuit elements [19, 20]. Blood pressure *P*(mmHg) corresponded to voltage, and flow rate *Q* (mL/s) was analogous to the current. Compliance of the artery played the role of capacitances *C* (mL/mmHg). *R* (mmHg·s/mL) and *L* (mmHg·s^2^/mL) represented impedance and inertia of the blood flow, respectively [20–22]. Based on the above assumptions, the cardiovascular system was depicted by the electrical circuit shown in Figure 2. 

### 2.1. Model of the Heart

An elastic model was defined to predict blood pressure of the left ventricular given as follows:

(1) $$\begin{array}{c} P_{\text{lv}}\left(t\right)=E_{\text{lv}}\left(t\right)*\left(V_{\text{lv}}-V_{d}\right)+P_{\text{th}}, \end{array}$$

where   *V*_lv_ (mL) is the stressed ventricular volume and *V*_*d*_ (mL) is a constant which is referred to as the ventricular volume at zero diastolic pressure. *P*_th_ (mmHg) stands for the intrapleural pressure. *E*_lv_ (mmHg/mL) represents the time-varying elasticity of the left ventricular.

Elastance-based model of the ventricles had been widely adopted since firstly proposed by Suga et al. in the 1970s [23, 24]. In this study, the idealized time evolution of the elastance function was used as follow [20]:

(2) $$\begin{array}{c} E_{\text{lv}}\left(t\right)=\{\begin{array}{cc} \frac{1}{2}\left(\frac{1}{\text{C}{\text{l}}_{\text{es}}}-\frac{1}{\text{C}{\text{l}}_{\text{ed}}}\right) & \\ \;*\left(1-\cos\left(\frac{\pi \left(t-t_{i}\right)}{T_{s}}\right)\right), & \\ \;\;\;\;\;\;\;\;t_{i}\leq t\leq t_{i}+T_{s}, & \\ \frac{1}{2}\left(\frac{1}{\text{C}{\text{l}}_{\text{es}}}-\frac{1}{\text{C}{\text{l}}_{\text{ed}}}\right) & \\ \;*\left(1+\cos\left(\frac{2\pi \left(t-\left(t_{i}+T_{s}\right)\right)}{T_{s}}\right)\right), & \\ \;\;\;\;\;\;t_{i}+T_{s}\leq t\leq t_{i}+T_{s}+T_{r}, & \\ \frac{1}{\text{C}{\text{l}}_{\text{ed}}},\;\;\;\;t_{i}+T_{s}+T_{r}\leq t\leq t_{i+1}, & \end{array} \end{array}$$

where the subscript *i* refers to the *i*th cardiac cycle. Cl_es_ and Cl_ed_ are values of end-systolic compliance and end-diastolic compliance, respectively. Furthermore, *T*_*s*_ and *T*_*r*_ respectively, stand for the systolic time period and the time for isovolumetric relaxation, which are functions of the cardiac cycle *T* (s):

(3) $$\begin{array}{c} T_{s}=0.3\sqrt{T},\;\;T_{r}=\frac{T_{s}}{2}=0.3\frac{\sqrt{T}}{2}. \end{array}$$

Values of the parameters used in the heart model mentioned above (Cl_ed_, Cl_es_, *V*_*d*_, *P*_th_ and *T*) are listed in Table 2.

### 2.2. Model of the Cardiac Valves

Cardiac valves played an important role in the cardiovascular system to ensure the blood flowing in the correct direction. A time-varying resistance model was developed to simulate the effect of the valves, which controlled the blood flow into (the mitral valve) and out of (the aortic valve) the left ventricle and it was described as [16, 25]:

(4) $$\begin{array}{c} R_{\text{mv}}=\min\left(R_{\text{mv},\text{open}}+\exp\left(-2\left(P_{\text{ve}}-P_{\text{lv}}\right)\right),20\right),\\ R_{\text{av}}=\min\left(R_{\text{av},\text{open}}+\exp\left(-2\left(P_{\text{lv}}-P_{\text{a}}\right)\right),20\right), \end{array}$$

where *P*_lv_, *P*_ve_, and *P*_a_ stand for the blood pressure of the left ventricle, the system vena, and aorta, respectively. *R*_mv,open_/*R*_av,open_ represents the baseline resistance value (seen in Table 2) when the mitral/aortic valve is opened. Accordingly, a small resistance is defined to depict the “open” valve, and a several orders larger resistance is used to simulate the “closed” valve.

### 2.3. Model of the Blood Vessel

**Figure 3:**
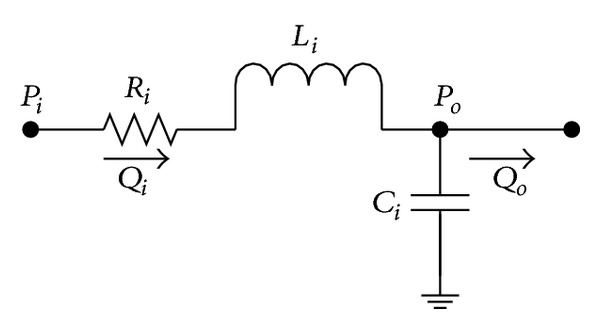
Electric circuit analog of the blood vessel segment. Flow through the model is defined by *Q* (mL/s). Pressures related to each compartment are marked by *P* (mmHg). Resistors are denoted by *R* (mmHg·s/mL), while capacitors and inductances are denoted by *C* (mL/mmHg) and *L* (mmHg·s^2^/mL), respectively.

In this model, an electrical circuit composed of linear electric elements was used to depict the cardiovascular system. For each of the arterial and venous units, the electric circuit model of the vascular was simulated as in Figure 3.

Differential equations were obtained by formulating the mass and momentum conservations, as follows:

(5) $$\begin{array}{c} \frac{dP_{o}}{dt}=\frac{Q_{i}-Q_{o}}{C},\\ \frac{dQ_{i}}{dt}=\frac{P_{i}-P_{o}-Q_{i}*R}{L}, \end{array}$$

where *Q*_*i*_ and *Q*_*o*_ are inflow and outflow of the related vessel, respectively. Similarly, *P*_*i*_ and *P*_*o*_ are blood pressure upstream and downstream of the related vessel, respectively.

Blocked blood vessels with various stenosis severities were simulated in order to account for the correlation between vascular stenoses and ABI. The stenosis severity *α* was defined as the percentage reduction in cross-sectional area of the related vessel [26] as follows:

(6) $$\begin{array}{c} \alpha =\left(1-\frac{A_{s}}{A_{0}}\right)\times 100\%, \end{array}$$

where *A*_*s*_ and *A*_0_ refer to cross-sectional areas of the stenotic and normal vessel segments. 

### 2.4. Solution of the Model

The governing differential equations of the model were solved with the fourth-order Runge-Kutta algorithm. Simulations started from early systole when the ventricles began to contract. The cardiac cycle was set to be 0.8 s, and physiological conditions were fixed for all of the simulations. The geometrical parameters of the 17 arterial units were prescribed based on the data reported in [27, 28] (Table 1). Values of the resistance (*R*), capacitance (*C*), and inductance (*L*) for each artery were calculated using (7) [27, 29] based on the geometrical data as follows:

(7) $$\begin{array}{c} R=\frac{8\mu l}{\pi r^{4}},\;\;C=\frac{3\pi lr^{3}}{2\text{Eh}},\;\;L=\frac{9\rho l}{4\pi r^{2}}, \end{array}$$

where *l*, *r* and *h* represent the length, radius and thickness of the vessel, respectively; *E* is the elastic parameter of the vascular; *ρ* is the blood density; *ν* is the viscosity of the blood. The values of *ρ* and *ν* are set as 1.06 g/mL and 0.004 Pa·s respectively, in this study.

### 2.5. Sensitivity Analysis of the Model

To better understand the stability of the parameters in the model, the sensitivity of the model output vectors on each of the parameters was analyzed. Basic methods for differential equation analysis [30, 31] were used to obtain the sensitivity equations of our cardiovascular system model. Following the algorithm developed by Ellwein et al. [16], the relative sensitivity *S*_*i*,*j*_ of the output vector *y*_*i*_ to the parameter *β*_*j*_ was defined by

(8) $$\begin{array}{c} S_{i,j}=\frac{\partial y_{i}}{\partial \beta_{j}}\frac{\beta_{j}}{y_{i}}{|}_{\beta =\beta_{0}},\;\beta_{j},y_{i}\neq 0, \end{array}$$

where *y*_*i*_ denotes the output vectors of the models, and here it refers to *P*_abl_/*P*_abr_, *P*_arl_/*P*_arr_, *P*_afl_/*P*_afr_, and *P*_atl_/*P*_atr_ (blood pressure of left/right brachial, radial, femoral, and posterior tibial artery, resp.) which could be detected noninvasively; *β*_*j*_ denotes all the parameters in the model (resistances, capacitances and inductances); *β*_0_ denotes the nominal values for the parameters. All of the variables are assumed to be continuous. Thus, *S*_*i*,*j*_ could be expressed as a function of time.

Based on the above computations, an averaged relative sensitivity *S*_*i*_ was used to get the total sensitivity to the *j*th parameter *β*_*j*_ in the following form [16, 32]:

(9) $$\begin{array}{c} S_{i}=\frac{1}{N}\sum_{j=1}^{N}|S_{i,j}|. \end{array}$$

## 3. Results

### 3.1. Validation of the Model

**Figure 4:**
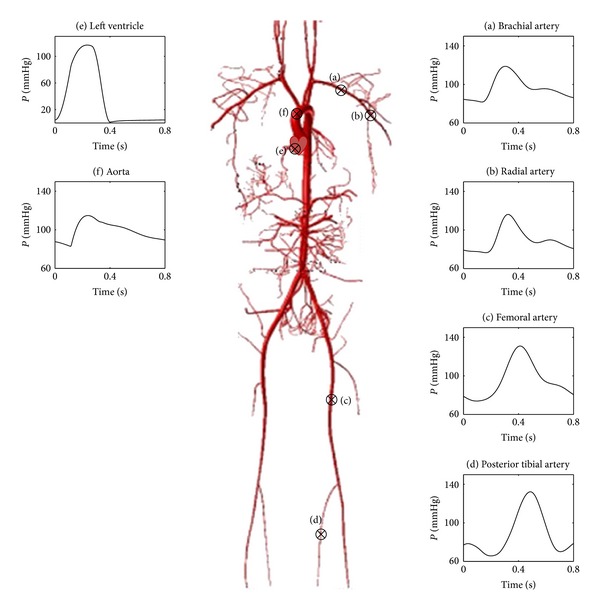
Blood pressure waveforms of the cardiovascular system in a single cardiac cycle.

Blood pressure pulses at several typical sites of the cardiovascular system in normal cases were shown in Figure 4. On the whole, the pressure waveforms obtained by the model were similar to the vivo data reported by Sun et al. [33], Reymond [34], and Blanco et al. [35]. The pressure magnitudes and time-constants involved were reasonable, thus verifying that the results obtained by the simulation model were consistent with human physiological conditions. 

To assess the sensitivity of the parameters, the indice *S* could be ranked into four classes as shown in Table 3 [32, 36]. The computational result showed that sensitivities of parameters in our model were mostly in grades I and II; only two parameters (*R*_mv,open_ and *R*_av,open_) were in grade III; thus, validated that our model was stable to simulate the pulse wave propagation in the cardiovascular system.

### 3.2. Sensitivity and Effectiveness of ABI

The computational model was applied further to study the influences on ABI of arterial stenoses located in femoral artery (no. 3), popliteal artery (no. 4), posterior tibial artery (no. 5), brachial artery (no. 11), radial artery (no. 12), and ulnar artery (no. 13), respectively. Moreover, the variation of ABI during the increase of the stenosis severity was also studied.

#### 3.2.1. Correlation between ABI and Stenosis Locations

**Figure 5:**
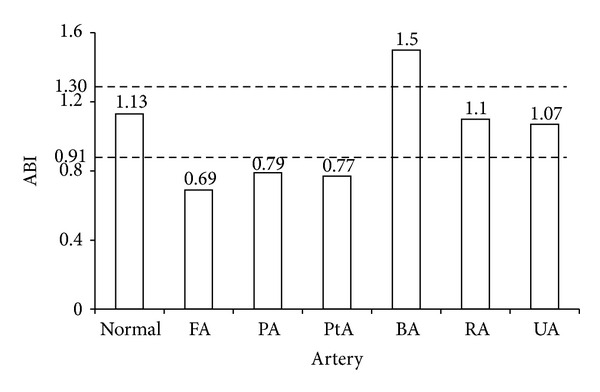
ABI for the normal case and for the stenosis cases with severities of 70% at 6 arteries, respectively. FA (femoral artery), PA (popliteal artery), PtA (posterior tibial artery), BA (brachial artery), RA (radial artery), and UA (ulnar artery) denote the locations of the stenoses. The dotted lines represent the evaluation standards of normal ABI (0.91~1.30) proposed by ACC/AHA. Vascular stenoses may have occurred if the value of ABI exceeds this range.

**Figure 6:**
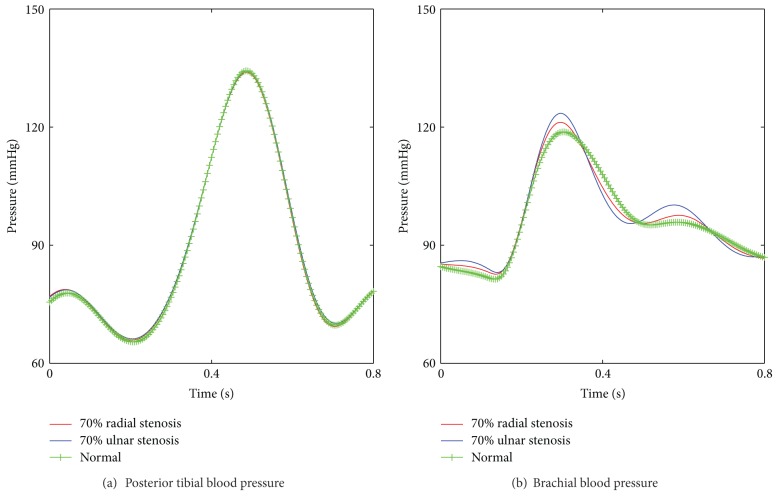
Comparison of the arterial blood pressure between the normal case and the cases with 70% radial stenosis and 70% ulnar stenosis respectively. (a) Posterior tibial blood pressure; (b) brachial blood pressure.

This model was used to study influences on ABI of stenoses located in different arteries. The simulation result (Figure 5) showed that ABI was capable to diagnose arterial stenoses in lower extremity arteries (femoral artery, popliteal artery, and posterior tibial artery). However, there were limitations to detect arterial stenoses in upper limb, other than brachial artery whose blood pressure directly affected ankle-brachial index calculations. It was difficult for ABI to detect radial and ulnar arterial stenoses for the reason that the relative decreases of brachial and posterior tibial systolic pressure were unconspicuous, as shown in Figure 6, which directly affected the result for ABI calculation in such vascular stenosis circumstances.

#### 3.2.2. Tendency of ABI with Arterial Stenosis of Increasing Severity

**Figure 7:**
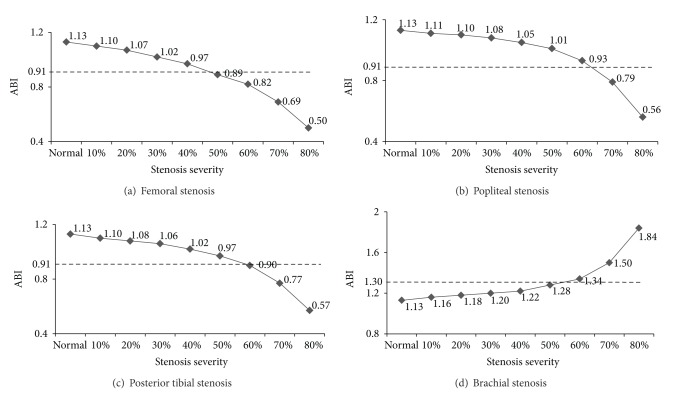
Changes of ABI value with stenosis increased from 0% to 80% at 4 arteries, respectively. (a) variation tendency of ABI with femoral stenosis; (b) variation tendency of ABI with popliteal stenosis; (c) variation tendency of ABI with posterior tibial stenosis; (d) variation tendency of ABI with brachial stenosis. The dotted line in each figure represents the evaluation standards of normal ABI (0.91~1.30) which is used as the threshold for stenoses diagnosis in clinical data.

**Figure 8:**
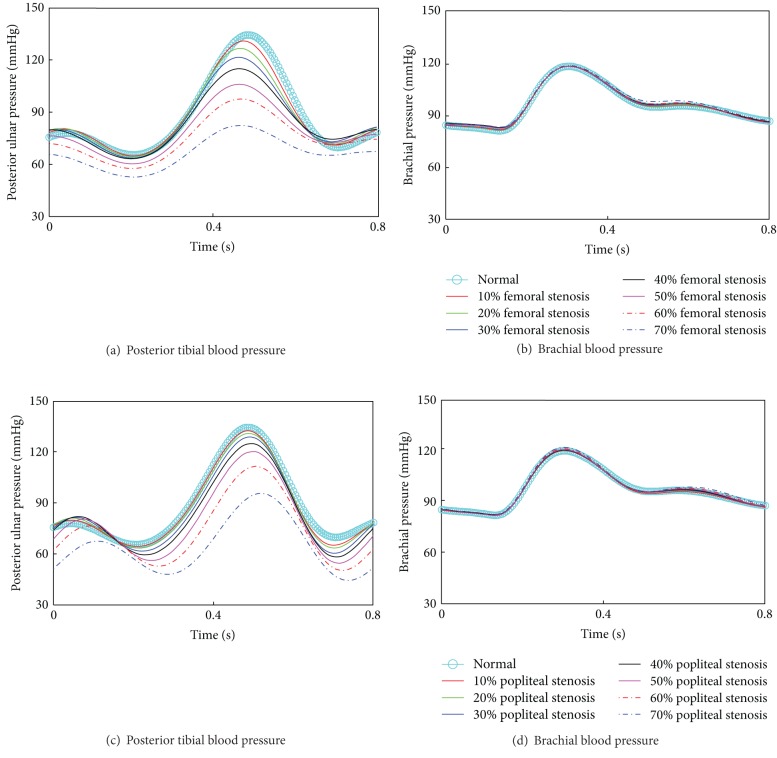
Blood pressure in a single cardiac cycle of posterior tibial artery and brachial artery with stenosis severities increasing from 0% to 70%. (a), (b): Stenoses located in femoral artery; (c), (d) Stenoses located in popliteal artery.

As ABI was effective to detect arterial stenoses of lower extremity limb arteries as well as brachial artery, assessment of the tendency of ABI with arterial stenoses of different severities was studied. The arterial stenosis was graded into three levels in clinical data: mild stenosis (1%~29%), moderate stenosis (30%~69%), and severe stenosis (70%~99%). As Figure 7 showed, the decrease/increase of ABI became larger when severity of stenoses was located in lower/upper limb artery changing from mild to severe. This was because systolic blood pressures in brachial artery changed more dramatically than those in posterior tibial artery in various arterial stenoses circumstances as shown in Figure 8. That was to say, ABI was more sensitive to severe stenoses than to mild/moderate stenoses located in all of the four arteries, both the lower extremity arteries (femoral artery, popliteal artery and posterior tibial artery) and the upper limb artery (brachial artery). 

## 4. Discussion

In the complex cardiovascular system, the hemodynamic influences of an arterial stenosis were closely related to the whole system. It was of great significance to detect arterial stenoses effectively and noninvasively for both the clinical diagnosis and the medical research. ABI, as a useful and a noninvasive detection method in clinical data, was proved to be effective and reliable in diagnosing arterial stenoses, especially for lower extremity arterial stenoses. Therefore, it was important to investigate the mechanism of ABI in detecting stenoses located in different sites of the cardiovascular system with various severities. Liang et al. [26] proposed that ABI was only effective for the stenosis present in the artery located in series with the ankle artery but parallel with the brachial artery. However, their research was based on study of stenoses located in aortic valvular, thoracic aorta, abdominal aorta, and so forth, without more analysis of stenoses located just in upper or lower limb arteries. There was little specific research about the distinction between diagnoses of stenoses located in lower limb arteries and in upper ones using ABI. The present study improved their study by constructing a computational multibranch model of the entire cardiovascular system clearly with several typical independent arterial units of upper and lower limbs.

Simulation results for the stenoses in six arteries showed that the ability of ABI to diagnose arterial stenoses depended strongly on the location where the stenosis occurred. ABI, as an index for assessing vascular stenoses, was effective for stenoses in lower extremity arteries (femoral artery, popliteal artery, and posterior tibial artery) and also brachial artery. However, the value of ABI was not able to predict stenoses of other upper limb arteries, such as radial and ulnar arterial stenoses. This was because the relative decreases of brachial and posterior tibial systolic pressure were unconspicuous (Figure 6), which directly affected the result for ABI calculation. It should be noted that there were limitations for ABI to predict brachial stenoses. The value of ABI could be more than 1.30, the upper bound of ABI, under the condition of severe brachial stenoses. Other imaging examination methods should be supplemented for the confirmation of brachial stenosis.

Our study also evidenced that the sensitivity and the effectiveness of ABI were higher to severe stenoses than to mild/moderate ones. This was because the changes of systolic blood pressures in brachial artery and posterior tibial artery that resulted from stenoses located in various lower limb arteries were different. Taking femoral arterial stenoses and popliteal arterial stenosis, for example (Figure 8), which did not affect the ABI calculation directly, the systolic blood pressure of posterior tibial artery was more sensitive than that of brachial artery to lower limb stenoses. Blood pressure of the posterior tibial artery changed more greatly when the stenosis severity increased from mild to severe. Accordingly, the variation of the ABI value became dramatical when it was calculated using the systolic blood pressure of posterior tibial artery divided by the systolic blood pressure of brachial artery. Therefore, the ABI, as a valid stenosis indicator, was more sensitive and effective to severe stenoses than to mild/moderate ones. The results were somehow supported by the clinical experimental data collected by Xu [15].

The present study was based on simulations for the cardiovascular system of a healthy adult without heart disease or other arterial diseases. However, the reality would be much more complicated by the idea that some changes of hemodynamic factors associated with a single stenosis in the study could be also caused by the presence of multiple arterial stenoses or other cardiac diseases. Furthermore, the variations of blood pressure might be underestimated, since the compensatory responses of the physiologic system to arterial stenoses were not considered. These limitations pointed to our future research, but they did not challenge the fundamental conclusions about the sensitivity and the effectiveness of ABI in detecting a single stenosis. 

## 5. Conclusion

A lumped parameter multibranch model of the cardiovascular system including 17 arterial units based on first-order differential equations has been developed in this study. This computational model has explicitly accounted for the pulse wave propagation in the arterial system. Furthermore, the model includes a physiological description of dynamics as a response to hemodynamic pressure changes caused by vascular stenoses and clearly depicts typical characteristic changes of the blood pressures. In clinical data, ABI is a useful, reliable and noninvasive method in detecting arterial stenoses, especially in diagnosing stenoses in lower extremity arteries. The model is thus applied to study the correlation between the stenosis and ABI.

Results show a strong location-dependence of ABI in predicting the stenosis. Stenoses located in the four arteries (femoral artery, popliteal artery, posterior tibial artery, and brachial artery) would cause significant variations of blood pressure in brachial and posterior tibial arteries, thus made ABI effective in diagnosing stenoses in these arteries. The main accomplishment of this study provides a theoretical basis for clinical diagnosis. It is also validated that ABI is more sensitive to severe stenoses than to mild/moderate ones, which is supported by the clinical experience of ABI diagnosis. The main accomplishment of this study has revealed the reason for why ABI acts as an effective index for arterial stenoses detection, providing the theoretical basis for optimizing the application of ABI. 

## Figures and Tables

**Table 1:** Physiologic geometry data of main arteries in the model.

No.	Arterial unit	Length *l *(cm)	Radius *r *(cm)	Thickness *h *(cm)	Elasticity *E* (∗10^6^ dyne/cm)
1	Brachial artery	23.5	0.2575	0.0525	4
2	Radial artery	23.4	0.1600	0.0430	8
3	Ulnar artery	23.7	0.1970	0.0470	8
4	Femoral artery	35.4	0.2400	0.0500	5
5	Popliteal artery	18.8	0.2000	0.0485	6
6	Posterior tibial artery	32.2	0.1800	0.0450	16
7	Anterior tibial artery 1	2.5	0.1300	0.0390	16
	Anterior tibial artery 2	30.0	0.1000	0.0200	16
8	Peroneal artery	31.8	0.1300	0.0290	16
9	Thoracic aorta	15.6	0.9830	0.1173	4
10	Abdominal aorta	15.9	0.6700	0.0893	4

**Table 2:** Values of the parameters used in the heart model and the cardiac valve model [16–18].

No.	Parameter	Value	Unit
1	Cl_ed_ (end-diastolic compliance of the left ventricle)	10	mL/mmHg
2	Cl_es_ (end-systolic compliance of the left ventricle)	0.4	mL/mmHg
3	*P*_th_ (intrapleural pressure)	−4	mmHg
4	*V*_*d*_ (ventricular volume at zero diastolic pressure)	10	mL
5	*R*_mv,open_ (resistance value of the open mitral valve)	0.014	mmHg·s/mL
6	*R*_av,open_ (resistance value of the open aortic valve)	0.006	mmHg·s/mL
7	*T* (cardiac cycle)	0.8	s

**Table 3:** Sensitivity classes.

Class	Index	Sensitivity
I	0.00 ≤ |*S* | <0.05	Small to negligible
II	0.05 ≤ |*S* | <0.20	Medium
III	0.20 ≤ |*S* | <1.00	High
IV	|*S* | ≥1.00	Very high
